# Examination of the effects of *Campylobacter concisus* zonula occludens toxin on intestinal epithelial cells and macrophages

**DOI:** 10.1186/s13099-016-0101-9

**Published:** 2016-05-18

**Authors:** Vikneswari Mahendran, Fang Liu, Stephen M. Riordan, Michael C. Grimm, Mark M. Tanaka, Li Zhang

**Affiliations:** School of Biotechnology and Biomolecular Sciences, University of New South Wales, Kensington, Sydney, NSW 2052 Australia; Gastrointestinal and Liver Unit, The Prince of Wales Hospital, Sydney, Australia; Prince of Wales Clinical School, University of New South Wales, Sydney, NSW 2052 Australia; St George and Sutherland Clinical School, University of New South Wales, Sydney, NSW 2052 Australia

**Keywords:** Zonula occludens toxin, *Campylobacter concisus*, Oral *Campylobacter* species, Inflammatory bowel disease

## Abstract

**Background:**

*Campylobacter concisus* is a Gram-negative bacterium that is associated with inflammatory bowel disease (IBD). Some *C. concisus* strains carry zonula occludens toxin (*zot*) gene which has polymorphisms. This study investigated the effects of *C. concisus* Zot on intestinal epithelial cells and macrophages using cell line models.

**Methods:**

*Campylobacter concisuszot*^808T^ gene, a polymorphism that is associated with active IBD, was cloned and expressed in *Escherichia coli*. The effects of *C. concisus* Zot on intestinal epithelial barrier were examined using Caco-2 cell model. Apoptosis induced by *C. concisus* Zot in Caco-2 cells was assessed by measuring the levels of caspase 3/7. The production of pro-inflammatory cytokines induced by *C. concisus* Zot in HT-29 cells and in THP-1 macrophage-like cells was measured using ELISA kits. Whether exposure to *C. concisus* Zot can affect the responses of macrophages to *E. coli* K12 was also investigated.

**Results:**

*Campylobacter concisus* Zot caused prolonged intestinal epithelial barrier damage, induced intestinal epithelial cell apoptosis, induced epithelial production of TNF-α and IL-8 and upregulated TNF-α in THP-1 macrophage-like cells. Pre-exposure to *C. concisus* Zot significantly enhanced the production of TNF-α and IL-8 as well as phagocytosis by THP-1 macrophage-like cells in response to *E. coli* K12.

**Conclusion:**

This study suggests that *C. concisus* Zot may have enteric pathogenic potential by damaging intestinal epithelial barrier, inducing intestinal epithelial and macrophage production of proinflammatory cytokines in particular TNF-α and enhancing the responses of macrophages to other enteric bacterial species.

**Electronic supplementary material:**

The online version of this article (doi:10.1186/s13099-016-0101-9) contains supplementary material, which is available to authorized users.

## Background

*Campylobacter concisus* is a Gram-negative bacterium that is associated with inflammatory bowel disease (IBD). A number of studies have detected a significantly higher prevalence of *C. concisus* in faecal samples and intestinal biopsies from patients with IBD as compared to controls [1–4]. The human oral cavity is the natural colonization site of *C. concisus* [5, 6]. However, *C. concisus* may colonize the intestinal tract in some individuals. Studies have shown that there are no distinct oral or enteric *C. concisus* strains and that *C. concisus* colonizing the human intestinal tract has originated from the individual’s own oral cavity or oral *C. concisus* strains from other sources [7, 8]. Previous studies suggested that some oral *C. concisus* strains have the potential to cause enteric disease [7, 9, 10]. In addition to patients with IBD, *C. concisus* was also frequently isolated from stool samples of patients with diarrheal disease [11–14].

A number of studies have identified potential virulence factors in *C. concisus* strains isolated from patients with IBD and in some instances healthy individuals [13, 15–17]. A study by Istivan et al. characterised and showed the effects of *C. concisus* phospholipase A on Chinese hamster ovary cells [15]. The other potential virulence factors in *C. concisus* and their abilities to cause any pathogenic changes to human cells remain to be investigated. One of such potential virulence factor is *C. concisus* zonula occludens toxin (*zot*) gene. Previously we detected the *zot* gene in 30 % of oral *C. concisus* strains and *C. concisus zot*^808T^ polymorphism was found to be associated with active IBD [17].

The *zot* gene was first detected in *Vibrio cholerae* and is encoded by CTX prophage [18]. The N terminus of *V. cholerae* Zot is involved in CTXφ morphogenesis while the C terminus is cleaved and secreted into the intestinal lumen [19]. The C terminal-fragment of *V. cholerae* Zot activates an intracellular signalling pathway by binding to proteinase activated receptor-2, which increases intestinal epithelial permeability by affecting the tight junctions [19].

*Campylobacter concisus zot* is carried by prophage CON_phi2 which is different from *V. cholerae* CTX prophage [20]. *Campylobacter concisus Zot* and *V. cholerae* Zot have only 16 % amino acid identity. To date, the biological effects of *C. concisus* Zot on human cells have not been investigated. In this study, the effects of *C. concisus* Zot on intestinal epithelial integrity, the phagocytic capacity of macrophages and production of pro-inflammatory cytokines in intestinal epithelial cells and macrophages were investigated using cell line models. The results from this study suggest that *C. concisus* Zot may have enteric virulent properties.

## Methods

### Expression of *C. concisuszot*^808T^ gene in *Escherichia coli* system

The full length *C. concisuszot*^808T^ gene was amplified from the *C. concisus* strain P14UCO-S1 by polymerase chain reaction (PCR) [17]. The PCR primers used for amplification are listed in Table 1. The amplified *zot* gene was cloned into plasmid vector pETBlue-2 with 6-histidines tagged at the C-terminus and expressed using a commercially available *E. coli* expression system following the manufacturer’s instructions (Novagen, WI, USA). The *E. coli* strain used for recombinant protein expression was BL21 (DE3) pLacI.

**Table 1:** Sequences of primers used for *zot* gene cloning

Primers	Sequence (5′–3′)	Reference
F_pETB2_P14UCO-S1	CACATG –*CCATGG*-CG **ATGTTAAGTTTGATTATAGGTCCG**	This study
R_pETB2_P14UCO-S1	CAATAA –*CTCGAG*^-**CTTGAGAGTAGGAAGCATAGG**	

Extra base pairs on the 5′ end of the primer are underlined and denote leader sequences, sequences in italics denote restriction enzyme sites for *NcoI*, sequences in italics and the symbol^ denote restriction enzyme site for *XhoI*, sequences in bold denote *zot* gene sequences

Nickel bound (Ni–NTA) agarose beads were used for partial purification of the expressed *C. concisus* Zot (Gold biotechnology, Inc., MO, USA). Proteins eluted from the Ni–NTA columns contained both the Zot protein of P14UCO-S1 and *E. coli* proteins (EP). These proteins were termed as EP-ZotP14UCO-S1. In order to ensure that the effects observed were due to *C. concisus* Zot proteins and not EP proteins, EP proteins were prepared by transforming *E. coli* BL21 (DE3) pLacI cells with pETBlue-2 vector; inducing the *E. coli* bacteria and purifying the proteins using identical protocols as for the purification of EP-ZotP14UCO-S1. The EP proteins were included in all experiments. All the experimental data from EP-ZotP14UCO-S1 were compared with that from the EP proteins.

The proteins eluted from Ni–NTA columns were filtered through 0.22 μm filter and then concentrated and buffer-exchanged to DPBS using an Amicon^®^ Ultra 10K column (Merck Millipore Ltd, Carrigtwohill, Ireland). The total protein concentrations were determined using a BCA assay kit (Thermo Fisher Scientific, MA, USA). The presence of *C. concisus* Zot in EP-ZotP14UCO-S1 was confirmed using anti-histidine antibody (Qiagen, Hilden, Germany) by Western blot as previously described [21].

### Examination of the effects of *C. concisus* Zot on intestinal epithelial barrier

Colorectal adenocarcinoma Caco-2 cells develop a monolayer of cells which express several morphological and functional characteristics that are similar to mature human enterocytes including assembly of the tight junctions, which have been extensively used as a model of intestinal epithelial barrier [22–24]. *Campylobacter concisus* was shown to damage intestinal epithelial barrier using Caco-2 cell model previously [25]. In this study, whether *C. concisus* Zot causes damage to intestinal barrier was investigated using Caco-2 cell model by examining the effects of EP and EP-ZotP14UCO-S1 proteins on transepithelial electrical resistance (TEER) and paracellular marker passage [26, 27]. Caco-2 cells were cultivated and maintained as previously described [7].

Caco-2 cells were grown on transwells with the pore size 0.4 μm for 21 days before utilization for TEER measurement (Millipore). TEER was measured using an Epithelial Volt/Ohm Meter (World Precision Instruments, FL, USA).

For TEER measurement, Caco-2 cells were incubated with 12.5 and 50 μg EP and EP-ZotP14UCO-S1 proteins dissolved in 100 μl DPBS respectively. Caco-2 cells incubated with DPBS were used as the negative control. Dimethyl sulfoxide (DMSO) (10 %), which is known to cause cell death, was used as the positive control for TEER measurement [28]. This positive control was to ensure that the damaged intestinal epithelial barrier is reflected by the decrease of TEER in our experimental conditions. The levels of TEER in Caco-2 cells treated with EP and EP-ZotP14UCOS1 proteins were expressed as a percentage relative to their time 0 TEER values respectively.

We aimed to examine both the short term effect and the prolonged effect of *C. concisus* Zot on TEER. For the short term effect, EP and EP-ZotP14UCO-S1 were incubated with Caco-2 cells for up to 4 h and TEER was measured at times 0, 2 and 4 h. The proteins and DPBS in the negative control cells were removed following 4 h of incubation in Caco-2 cells and the normal cell culture media was added. TEER was measured again at different time points after the removal of *C. concisus* Zot and this was regarded as the prolonged effect of *C. concisus* Zot on TEER.

Paracellular marker assay was carried out by using fluorescein-labelled dextran 4000 (FD4). Given that EP-ZotP14UCO-S1 (50 μg protein dissolved in 100 μl of DPBS) caused a significant decrease of TEER in Caco-2 cells after 2 h of incubation in the above experiment, the same time point was also used for the paracellular marker assay. Caco-2 cells were grown as mentioned above and were incubated with EP and EP-ZotP14UCO-S1 proteins. Caco-2 cells incubated with DPBS were used as the negative control and 10 % DMSO treated Caco-2 cells were used as the positive control [28]. Following 2 h of incubation, cells were washed with DPBS and 700 μg/ml of FD4 was added. The cells were incubated for further 2 h and the passage of FD4 was then measured. The permeability of FD4 was calculated as apparent permeability coefficient (Papp) value as previously described [26].

### Measurement of caspase 3/7 activity in Caco-2 cells induced by *C. concisus* Zot

Caspase 3/7 activity was measured in order to examine whether *C. concisus* Zot caused apoptosis in Caco-2 cells. Caco-2 cells were cultured on black-walled 96-well plates with transparent bottom for 2 days and then incubated with EP and EP-ZotP14UCO-S1 proteins at 50 μg/100 μl for 2 h. Caco-2 cells incubated in DPBS were used as the negative control and DMSO (5 %) treated Caco-2 cells were used as the positive control [29]. Caspase 3/7 activity was then measured using fluorescent CellEvent Caspase-3/7 Green ReadyProbes Reagent (Thermo Fisher Scientific) as per the manufacturer’s instruction [28]. The level of caspase 3/7 activity in Caco-2 cells treated with EP and EP-ZotP14UCO-S1 were expressed as folds relative to that in the negative control Caco-2 cells.

### Measurement of pro-inflammatory cytokines in HT-29 cells induced by *C. concisus* Zot

Whether *C. concisus* Zot can induce intestinal epithelial production of proinflammatory cytokines was investigated. We initially measured IL-8 and TNF-α in the supernatants of Caco-2 cells treated with EP and EP-P14UCOS1 for 8 and 24 h. However, the cytokine levels produced by Caco-2 cells in our experiments were undetectable. Given this, human colon carcinoma HT-29 cells, which are known to produce higher levels of pro-inflammatory cytokines, were used to examine the effects of EP and

EP-P14UCOS1 proteins on induction of intestinal epithelial production of proinflammatory cytokines [30–32]. A previous study showed that *C. concisus* strains induced apoptosis in HT-29 cells [9]. However, in this study we did not examine the apoptosis in EP and EP-P14UCOS1 treated HT-29 cells given that some proinflammatory cytokines are known to cause apoptosis.

HT-29 cells were cultivated and maintained as previously described [21]. HT-29 cells were treated with EP and EP-ZotP14UCO-S1 proteins (50 μg in 100 μl) for 24 h and supernatants were collected. The levels of TNF-α and IL-8 in the HT-29 culture media were quantitated using Human TNF-α and IL-8 CytoSet (Thermo Fisher Scientific). HT-29 cells incubated in DPBS were used as the negative control and *C. concisus* strain P1CDO3 at MOI 200 was used as the positive control [21]. The purpose of the positive control in this experiment was to ensure the cells used were able to elicit production of pro-inflammatory cytokines in response to bacteria or their products. P1CDO3 was shown to induce IL-8 production in HT-29 cells in our previous study, therefore was used as the positive control in this study [21].

### Measurement of proinflammatory cytokines in THP-1 macrophages-like cells induced by *C. concisus* Zot

THP-1 is a human monocytic cell line derived from an acute monocytic leukemia patient. Once treated with phorbol 12-myristate 13-acetate (PMA), THP-1 cells undergo greater differentiation to become macrophage-like cells and have increased adherence and expression surface markers, which have been frequently used a cell culture model for macrophages [33]. *Campylobacter concisus* was previously shown to induce the production of proinflammatory cytokines in THP-1 macrophage-like cells [25]. To examine whether *C. concisus* Zot have effects on macrophage functions, we examined the production of IL-8 and TNF-α production in THP-1 macrophage-like cells and the responses of THP-1 macrophage-like cells to other bacterial species following the incubation with EP and EP-P14UCOS1.

THP-1 macrophage-like cells were maintained as previously described [34]. PMA (10 nM) (Sigma-Aldrich, NSW, Australia) was added to the cells for 2 days to allow the differentiation of THP-1 cells to macrophage-like cells. The cells were then cultured in media without PMA for further 2 days prior to experiments. THP-1 macrophage-like cells (1 × 10^5^/well in 96-well plate) were incubated with EP and EP-ZotP14UCO-S1 proteins at 50 μg/100 μl for 2 h.

The supernatants were collected and the levels of TNF-α and IL-8 were measured as described above. THP-1 macrophage-like cells incubated in DPBS were used as the negative control and the resulting levels of cytokines were subtracted from the test samples. *Campylobacter concisus* strain P1CDO3 at MOI 200 was used as the positive control to ensure that THP-1 macrophage-like cells elicited production of pro-inflammatory cytokines.

### The effect of pre-exposure to *C. concisus* Zot on the phagocytic capacity of THP-1 cells to *E. coli* K12 and cytokine production

We are interested in examining whether exposure to *C. concisus* Zot affects the responses of macrophages to other enteric bacterial species. To investigate this, we examined the phagocytosis of THP-1 macrophage-like cells to *E. coli* K12 and cytokine production. Vybrant Phagocytosis Assay kit (Molecular Probes, Oregon, USA) was used to measure phagocytosis in EP and EP-ZotP14UCO-S1 proteins treated macrophages.

THP-1 macrophages like cells were incubated with EP and EP-ZotP14UCO-S1 proteins at 50 μg/100 μl for 2 h as described above. The controls were included as per the manufacturer’s instruction. The negative control for this assay constituted of microplate wells containing only media incubated with *E. coli* K12 Bioparticles. The positive control for this assay constituted of untreated THP-1 macrophages-like cells which were incubated with *E. coli* K12 Bioparticles. Post incubation, the proteins were removed and cells were washed.

Fluorescein-labelled *E. coli K12* BioParticles (100 μl) were subsequently added to the cells. Following 2 h of incubation, supernatant was removed and used for measurement of TNF-α and IL-8 as described above. The phagocytosed *E. coli* K12 BioParticles were measured using a Fluorescence Plate Reader as per the manufacturer’s instruction. The levels of phagocytosis in THP-1 macrophage-like cells treated with EP and EP-ZotP14UCO-S1 proteins were expressed as percentage relative to that of the positive control after subtraction from the value of the negative control.

### Statistical analysis

Data were analyzed by means of unpaired Student’s *t* test using GraphPad, Prism version 5.1 (San Diego, CA). *P* values less than 0.05 (two tailed, 95 % confidence interval) indicated statistical significance.

## Results

### The effect of different concentrations of EP-ZotP14UCO-S1 on TEER of Caco-2 cells

**Fig. 1:**
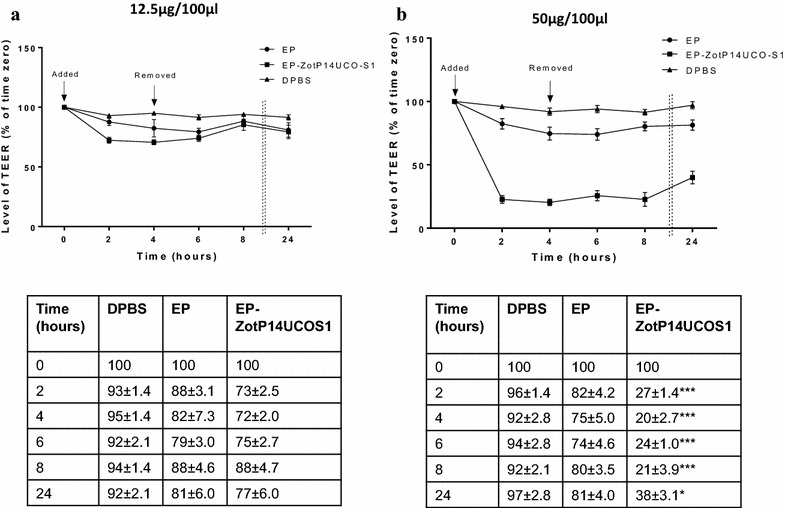
The effect of *C. concisus* Zot on TEER. Caco-2 cells grown for 21 days were treated apically with two concentrations (**a** 12.5 μg/100 μl, **b** 50 μg/100 μl) of EP and EP-ZotP14UCO-S1 proteins dissolved in DPBS. Caco-2 cells in DPBS were utilized as the negative control. Following 4 h of incubation, the proteins and DPBS were removed and cells were further incubated. TEER was measured at different time points. TEER was expressed as the percentage relative to TEER reading at time 0. *Dotted lines* indicate lapsed times between 8 and 24 h. The levels of TEER in Caco-2 cells treated with EP-ZotP14UCO-S1 and EP were compared. The TEER values for DMSO treated cells were 59 ± 4.0 %. * Indicates *P* < 0.05 and *** Indicates *P* < 0.001. Data are shown as the mean ± SD from triplicates and are representative of three independent experiments. EP-ZotP14UCO-S1: constituted of *E. coli* proteins and the Zot of *C. concisus* strain P14UCO-S1 expressed in *E. coli*. *EP* control *E. coli* proteins. *TEER* transepithelial electrical resistance

Two different concentrations of EP and EP-ZotP14UCO-S1 proteins were used to treat Caco-2 cells. When 12.5 μg of total proteins were used, a minor decrease in TEER was observed in Caco-2 cells treated with EP-ZotP14UCO-S1 as compared to EP treated Caco-2 cells following 2 and 4 h of incubation (Fig. 1a). However, TEER increased after removal of the proteins. When 50 μg total proteins were used, EP-ZotP14UCO-S1 caused a significant decrease of TEER as compared to EP treated cells following 2 and 4 h of incubation (Fig. 1b) (*P* < 0.001).

The TEER level measured at 24 h after the removal of the proteins in Caco-2 cells treated with EP-ZotP14UCO-S1 was still significantly lower than that in Caco-2 cells treated with EP (*P* < 0.05) (Fig. 1b). This showed that EP-ZotP14UCO-S1 had a prolonged effect on the TEER of Caco-2 cells. Given this, 50 μg of total proteins resuspended in 100 μl DPBS was used for the remaining experiments. DMSO treated cells caused TEER to drop to 59 ± 4.0 and 97 ± 2.8 % of its initial TEER after 2 and 24 h of incubation respectively. The TEER in Caco-2 cells incubated in DPBS remained stable through the course of the experiment.

### The effect of EP-ZotP14UCO-S1 on paracellular permeability in Caco-2 cells

**Fig. 2:**
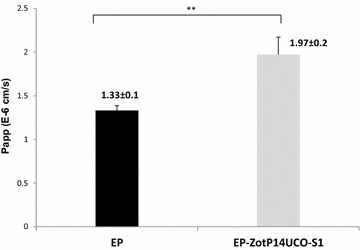
The effect of *C. concisus* Zot on the passage of paracellular marker. Caco-2 cells grown for 21 days were treated with 50 μg/100 μl of EP and EP-ZotP14UCO-S1 proteins for 2 h. Cells were then washed with DPBS and 700 μg/ml of fluorescein-labelled dextran 4000 (FD4) was added. The cells were further cultured for 2 h and the passage of FD4 was measured. The permeability of FD4 was calculated as apparent permeability coefficient (Papp). The Papp value caused by 10 % DMSO treated cells was 4.58 ± 0.1. Data are shown as the mean ± SD from triplicates and are representative of three independent experiments. ** Indicates *P* < 0.01. EP-ZotP14UCO-S1: constituted of *E. coli* proteins and the Zot of *C. concisus* strain P14UCO-S1 expressed in *E. coli*. *EP* control *E. coli* proteins

The effect of EP-ZotP14UCO-S1 on Caco-2 paracellular permeability was examined by using the paracellular marker FD4. The Papp value in Caco-2 cells treated with EP-ZotP14UCO-S1 was 1.97 ± 0.2 and this was significantly higher as compared to EP treated cells (1.33 ± 0.1, *P* < 0.01) (Fig. 2). This indicated that the decrease in TEER induced by EP-ZotP14UCO-S1 at 2 h directly correlated with an increase in paracellular permeability. The Papp value caused by 10 % DMSO treated cells was 4.58 ± 0.1.

### The effect of EP-ZotP14UCO-S1 on Caco-2 cells apoptosis

Caspase 3/7 levels were measured to examine for apoptosis induced by *C. concisus* Zot in Caco-2 cells. The caspase 3/7 activity was 2.53 ± 0.03 in Caco-2 cells treated with EP-ZotP14UCO-S1, and this was significantly higher than that in cells treated with EP (1.53 ± 0.1, *P* < 0.05). The caspase 3/7 levels in 5 % DMSO treated cells were 2.68 ± 0.4.

### The production of TNF-α and IL-8 in HT-29 cells induced by EP-ZotP14UCO-S1

**Fig. 3:**
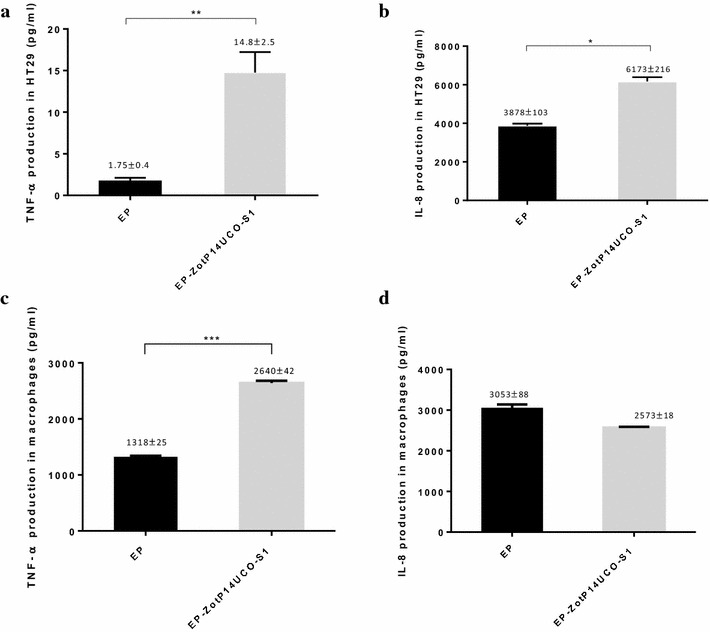
The effect of *C. concisus* Zot on the production of TNF-α and IL-8 in HT-29 and THP-1 macrophage-like cells. HT-29 cells (**a** and **b**) HT-29 cells were treated with EP and EP-ZotP14UCO-S1 proteins at 50 μg/100 μl for 24 h. The supernatants were collected and the levels of TNF-α and IL-8 were measured using commercially available ELISA kits. TNF-α and IL-8 levels in P1CDO3 treated cells were 34 ± 8 and 246 ± 27 pg/ml respectively. THP-1 macrophage-like cells (**c** and **d**) THP-1 macrophage-like cells were treated with EP and EP-ZotP14UCO-S1 proteins at 50 μg/100 μl for 2 h. The supernatants were collected and the levels of TNF-α and IL-8 were measured using commercially available ELISA kits. Data are shown as the mean ± SD from triplicates and are representative of two independent experiments. The value in the negative control (cells without any treatment) was subtracted from the data presented in this figure. TNF-α and IL-8 levels in P1CDO3 treated cells were 1738 ± 230 and 1708 ± 187 pg/ml respectively. *P < 0.05, **P < 0.01 and ***P < 0.001. EP-ZotP14UCO-S1: constituted of *E. coli* proteins and the Zot of *C. concisus* strain P14UCO-S1 expressed in *E. coli*. *EP* control *E. coli* proteins

The levels of TNF-α and IL-8 in EP-ZotP14UCO-S1 treated HT-29 cells were 14.8 ± 2.5 and 6173 ± 216 pg/ml respectively. These levels were significantly higher than those induced by EP treated cells (*P* < 0.05) (Fig. 3a, b). TNF-α and IL-8 levels in HT-29 cells incubated with *C. concisus* strain P1CDO3 were 34 ± 8 and 246 ± 27 pg/ml respectively.

### The production of TNF-α and IL-8 in THP-1 macrophage-like cells induced by EP-ZotP14UCO-S1

In THP-1 macrophage-like cells, EP-ZotP14UCO-S1 (2640 ± 42 pg/ml) induced a significantly higher production of TNF-α as compared to EP treated cells (1318 ± 25 pg/ml) (*P* < 0.05) (Fig. 3c). EP-ZotP14UCO-S1 treated cells did not induce a significant change in the production of IL-8 as compared to EP treated cells (Fig. 3d). TNF-α and IL-8 production in THP-1 cells incubated with *C. concisus* strain P1CDO3 were 1738 ± 230 and 1708 ± 187 pg/ml respectively.

### The effect of exposure to EP-ZotP14UCO-S1 on the responses of THP-1 macrophage-like cells to *E. coli* K12

**Fig. 4:**
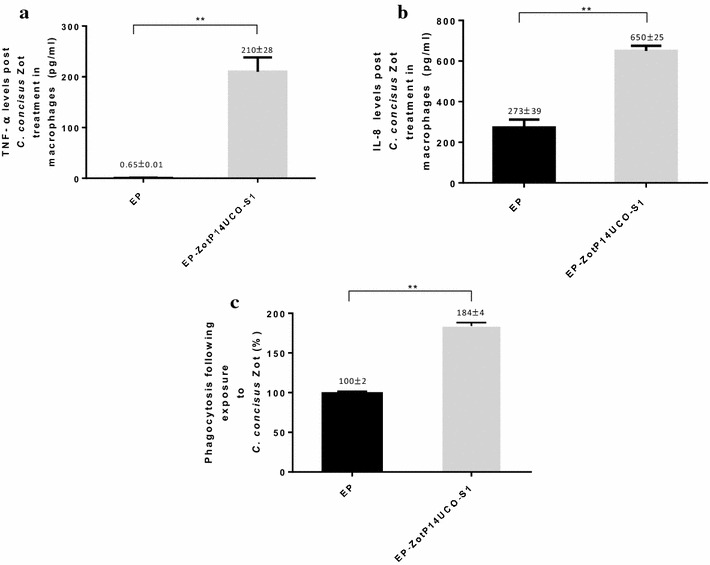
Exposure to *C. concisus* Zot enhances the response of THP-1 macrophage-like cells to *E. coli* K12. The production of pro-inflammatory cytokines and phagocytosis by THP-1 macrophage-like cells enhances the response to *E. coli* K12 following exposure to *C. concisus* Zot were assessed. THP-1 cells were treated with EP and EP-ZotP14UCO-S1 at 50 μg/100 μl for 2 h. The proteins were removed and cells were washed with DPBS. Measurement of cytokines levels (**a** and **b**) the cells were then incubated with *E.coli* K12 Bioparticles for an additional 2 h and the supernatants were used to measure TNF-α and IL-8 using commercially available ELISA kits. Phagocytosis (**c**) the phagocytosed *E. coli* K12 Bioparticles were measured using a Fluorescence Plate Reader. The values in the negative control were subtracted from the positive control and experimental wells. The levels of phagocytosis in THP-1 macrophage-like cells treated with different proteins were expressed as percentage relative to that of the positive control. Data are shown as the mean ± SD from triplicates and are representative of two independent experiments. ** Indicates *P* < 0.01. EP-ZotP14UCO-S1 constituted of *E. coli* proteins and the Zot of *C. concisus* strain P14UCO-S1 expressed in *E. coli*. *EP* control *E. coli* proteins

The levels of TNF-α and IL-8 levels induced by *E. coli* K12 in THP-1 cells without pre-exposure to *C. concisus* Zot were subtracted from the data present in Fig. 4a, b. Pre-exposure to EP for 2 h did not appear to affect the production of TNF-α in THP-1 cells in response to *E. coli* K12 (Fig. 4a). THP-1 cells produced significantly higher levels of TNF-α following pre-exposure to EP-ZotP14UCO-S1 (210 ± 28 pg/ml) when compared to EP treated cells (0.65 ± 0.01 pg/ml) (*P* < 0.01) (Fig. 4a). The levels of IL-8 produced by THP-1 cells in response to *E. coli* K12 following pre-exposure to EP-ZotP14UCO-S1 (650 ± 25 pg/ml) were also significantly higher when compared to EP (273 ± 39 pg/ml) (*P* < 0.05) (Fig. 4b).

### The effect of pre-exposure to *C. concisus* Zot on the phagocytic capacity of THP-1 cells to *E. coli* K12

The exposure to EP-ZotP14UCO-S1 (184 ± 4 %) for 2 h significantly increased the phagocytosis of *E. coli* by THP-1 cells when compared to cells exposed to EP treated cells (100 ± 2) (*P* < 0.01) (Fig. 4c).

## Discussion

In this study, we examined the effects of *C. concisus* Zot on intestinal epithelial cells and macrophages using cell line models.

EP-ZotP14UCO-S1 caused a significant decrease in TEER and an increase in paracellular permeability as compared to EP proteins in Caco-2 cell intestinal epithelial barrier model. Interestingly, the TEER level in Caco-2 cells treated with EP-ZotP14UCO-S1 remained significantly lower 24 h after the removal of the proteins (Figs. 1b, 2). The poor recovery of TEER levels after 24 h in EP-ZotP14UCO-S1 treated Caco-2 cells suggests that apoptosis had occurred in these cells. Indeed, EP-ZotP14UCO-S1 proteins caused a significant increase in caspase 3/7 levels in Caco-2 cells as compared to EP proteins, suggesting that apoptosis could be a possible mechanism contributing to the decreased TEER. Induction of epithelial apoptosis by EP-ZotP14UCO-S1 in Caco-2 cells suggests that *C. concisus* Zot may contribute to enteric disease through causing a prolonged damage to intestinal epithelial barrier. We have measured the levels of IL-8 and TNF-α in the supernatants of Caco-2 cells following incubation with EP and EP-ZotP14UCO-S1 for 8 and 24 h. However, the levels of these cytokines produced by Caco-2 cells were undetectable. These data suggest that the apoptosis induced by EP-ZotP14UCO-S1 in Caco-2 cells was due to a different mechanism rather than through the induction of TNF-α.

In addition to causing damage to the intestinal epithelial barrier in Caco-2 cells, EP-ZotP14UCO-S1 induced significantly higher production of TNF-α and IL-8 in HT-29 cells and a significantly higher production of TNF-α in the macrophages (Fig. 3a, b). TNF-α plays a central role in the pathogenesis of IBD [35]. The levels of TNF-α are raised in the serum, mucosa and stool of IBD patients [36–38]. Treatment using monoclonal anti-TNF antibody has shown to be a successful therapy in a subgroup of IBD [36–38]. Overproduction of TNF by intestinal epithelial cells was shown to cause full advancement of Crohn-like pathology in an animal model [39]. The increased elicitation of TNF-α by EP-ZotP14UCO-S1 in intestinal epithelial cells and macrophages suggests that *C. concisus* Zot may be a key microbial factor in initiating and contributing to the pathogenesis of IBD. However, this speculation requires further investigation.

Exposure to EP-ZotP14UCO-S1 significantly enhanced the responses of THP-1 macrophage-like cells to *E. coli* K12. This included increased production of pro-inflammatory cytokines and phagocytic activities (Fig. 4a–c). The molecular mechanism for this is not clear. One probable cause of this effect could be that TNF-α produced by macrophages during pre-incubation with EP-ZotP14UCO-S1 had activated the THP-1 macrophage-like cells [40]. The finding that exposure to EP-ZotP14UCO-S1 enhanced the responses of THP-1 macrophage-like cells to *E. coli* K12 suggests that *C. concisus* Zot has the potential to influence the responses of intestinal innate immune system to enteric resident bacterial species, which may contribute to chronic mucosal inflammation. Future studies should be conducted for further investigation.

Despite the interesting findings, this study is not without its own limitations. The expression level of *C. concisus* Zot in *E. coli* was low and thus partially purified Zot was used (Additional file 1: Figure 1A, B). Various experimental conditions were employed to increase the expression level of *C. concisus* Zot and to increase the purity of *C. concisus* Zot eluted from Ni–NTA columns, which yielded limited success. We noted that the growth of *E. coli* that were transformed with pETBlue-2-*zot*^808T^ceased following the induction of Zot expression and the *E. coli* bacteria appeared to be less healthy under microscope, as compared to *E. coli* bacteria that were transformed with pETBlue-2 vector alone. This suggests that *C. concisus* Zot may be toxic to host *E. coli* bacteria, which is a possible factor contributing to the low level expression of *C. concisus* Zot in *E. coli*. Changing imidazole and salt concentrations and inclusion of some detergents in the purification of *C. concisus* Zot did not further increase the purity of Zot protein eluted from Ni–NTA columns. Nevertheless, in this study we included the same concentrations of EP proteins in all experiments. EP-ZotP14UCO-S1 and EP proteins clearly had different effects on intestinal epithelial cells and macrophages. These results provide initial evidence that *C. concisus* Zot has the potential to affect the functions of intestinal epithelial cell and macrophages.

## Conclusion

In summary, using cell culture models we found that *C. concisus* Zot caused prolonged intestinal epithelial barrier damage, induced intestinal epithelial apoptosis and intestinal epithelial production of TNF-α and IL-8. Furthermore, *C. concisus* Zot upregulated the production of TNF-α in THP-1 macrophage-like cells and enhanced the responses of THP-1 macrophage-like cells to *E. coli* K12. These findings suggest that *C. concisus* Zot has the potential to cause enteric disease by damaging intestinal barrier, inducing intestinal epithelial and macrophage production of proinflammatory cytokines in particular TNF-α and enhancing the responses of macrophages to other enteric bacterial species.

## Additional file

10.1186/s13099-016-0101-9 SDS-PAGE and Western blot images of EP and EP-ZotP14UCO-S1 proteins. EP and EP-ZotP14UCO-S1 proteins were purified from *E. coli* transformed with pETBlue-2 vector or pETBlue-2-*zot* ^808T^ using Ni–NTA columns as per manufacturer’s instruction. The flow through, column wash and eluates were collected and subjected to SDS-PAGE to reveal proteins. Eluates were also subjected to Western blot to detect Zot using anti-histidine antibodies. A and B purification of EP, A showing SDS-PAGE and B showing Western blot. C and D purification of EP-ZotP14UCO-S1, C showing SDS-PAGE and D showing Western blot. The Zot protein (44 kD) was detected in EP-P14UCO-S1 not in EP. FT (Flow through), W1 (first wash), W2 (second wash), El-E4 (elutions 1-4). E1 was used for conducting experiments in this study.
