# Supplementary figures and images for: Examination of the effects of Campylobacter concisus zonula occludens toxin on intestinal epithelial cells and macrophages
Source: Gut Pathog. 2016 May 18;8:18. doi: 10.1186/s13099-016-0101-9 (PMC4870807; doi:10.1186/s13099-016-0101-9)

**A**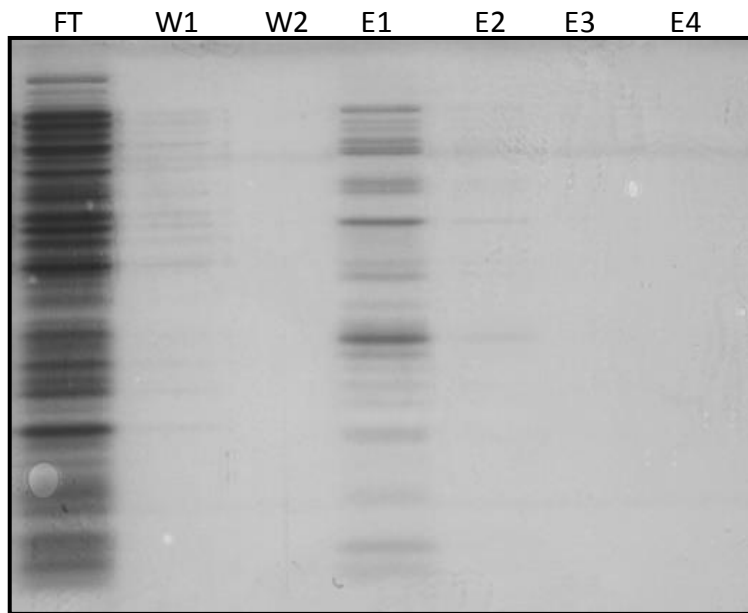**B**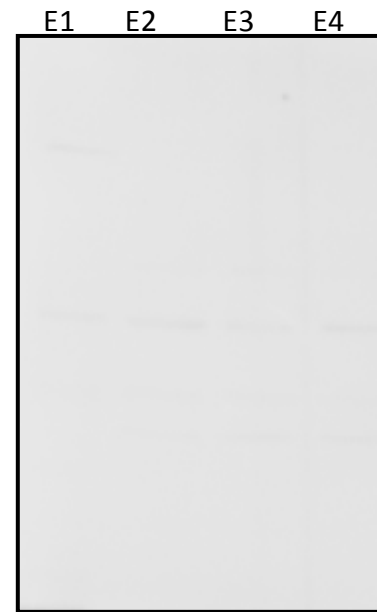**C**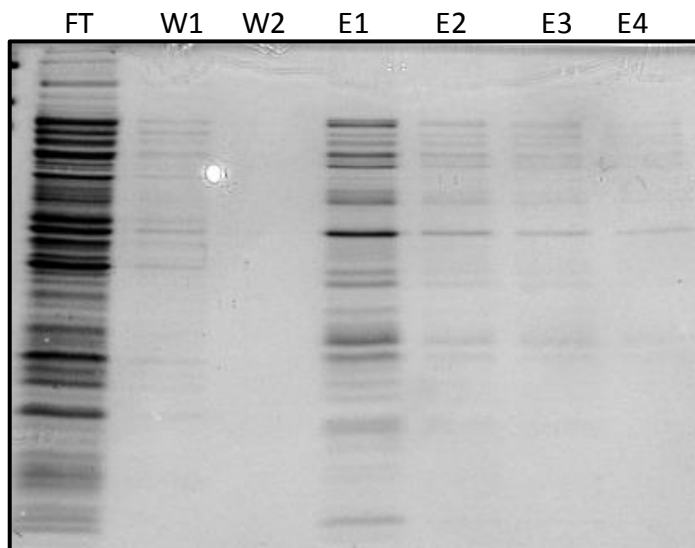**D**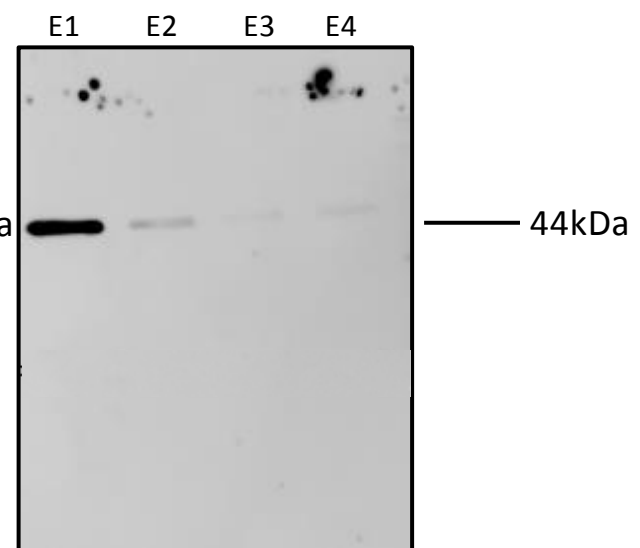

Supplement: Supplementary file 1 — 10.1186/s13099-016-0101-9 SDS-PAGE and Western blot images of EP and EP-ZotP14UCO-S1 proteins. EP and EP-ZotP14UCO-S1 proteins were purified from E. coli transformed with pETBlue-2 vector or pETBlue-2-zot 808T using Ni–NTA columns as per manufacturer’s instruction. The flow through, column wash and eluates were collected and subjected to SDS-PAGE to reveal proteins. Eluates were also subjected to Western blot to detect Zot using anti-histidine antibodies. A and B purification of EP, A showing SDS-PAGE and B showing Western blot. C and D purification of EP-ZotP14UCO-S1, C showing SDS-PAGE and D showing Western blot. The Zot protein (44 kD) was detected in EP-P14UCO-S1 not in EP. FT (Flow through), W1 (first wash), W2 (second wash), El-E4 (elutions 1-4). E1 was used for conducting experiments in this study. [file 13099_2016_101_MOESM1_ESM.pdf]
